# Engineering of *Rhodococcus jostii* RHA1 for utilisation of carboxymethylcellulose

**DOI:** 10.1016/j.heliyon.2023.e19511

**Published:** 2023-08-25

**Authors:** Rabia Yasin, Goran M.M. Rashid, Imran Ali, Timothy D.H. Bugg

**Affiliations:** a Department of Chemistry, University of Warwick, Coventry, CV4 7AL, UK; b Department of Biotechnology, Mirpur University of Science and Technology (MUST), Mirpur, 10250, AJK, Pakistan

**Keywords:** *Rhodococcus jostii* RHA1, Cellulose, Metabolic engineering, Endoglucanase, Exoglucanase, Quinic acid

## Abstract

*Rhodococcus jostii* RHA1 was engineered to utilise the cellulose component of lignocellulose, as well as the lignin fraction, by introduction of cellulase genes. The genome of *R. jostii* RHA1 was found to contain two β-glucosidase genes, RHA1_ro01034 and RHA1_ro02947, which support growth on cellobiose as growth substrate. Five Gram-positive endocellulase genes and one exocellulase gene were cloned into expression vector pTipQC2, and expressed in *R. jostii* RHA1. Endoglucanase activity was detected, with highest activity using *Cellulomonas fimi cenA*, and this recombinant strain grew on minimal media containing 0.5% carboxymethylcellulose (CMC). The *R. jostii* RHA1 genome was also found to contain a 3-dehydroshikimate dehydratase gene RHA1_ro01367, which supports growth on quinic acid as growth substrate, and conversion to protocatechuic acid. Therefore, this bacterium shows promise for further engineering to utilise cellulose for conversion to protocatechuic acid-derived bioproducts.

## Highlights

• *Rhodococcus jostii* RHA1 was engineered to utilise carboxymethylcellulose as growth substrate.
• Highest endoglucanase activity was detected using *Cellulomonas fimi cenA.*
• The *R. jostii* RHA1 genome contains a 3-dehydroshikimate dehydratase gene.
• *R. jostii* RHA1 can also grow on quinic acid as growth substrate.

## 1 Introduction

Bacteria in the Rhodococcus genus have found a range of applications in biotechnology, due to their ability to degrade a range of compounds, their resistance to toxicity, and ability to grow well under bioreactor conditions [1]. In particular, Rhodococci have found recent applications as microbial degraders of lignin, the aromatic heteropolymer found in lignocellulose plant cell walls. *Rhodococcus jostii* RHA1 is a polychlorinated biphenyl-degrading bacterium whose genome was sequenced in 2006 [2], and this strain was identified in 2010 as a lignin-degrading bacterium [3]. This bacterium has been used as a host for metabolic engineering approaches for lignin degradation, to generate vanillin [4] and pyridine-dicarboxylic acids [5,6] as bioproducts. *Rhodococcus opacus* strains PD630 and DSM 1069, which accumulate triacylglycerol lipids, have also been shown to convert organosolv lignins into lipids [7].

Although successful conversion of the lignin component of wheat straw lignocellulose to vanillin [4] and pyridine-dicarboxylic acids [5,6] by engineered *R. jostii* RHA1 strains has been achieved, only 5–15% of the lignin content was converted to products, therefore, there is a need to improve product titres and conversion yields. One approach to improve yield from lignocellulose feedstocks would be to engineer *Rhodococcus jostii* RHA1 to utilise the cellulose content of lignocellulosic biomass, via conversion to d-glucose. Glucose could then in principle be converted via the shikimate pathway to protocatechuic acid, a key intermediate in aromatic degradation, utilising the *asbF* gene encoding 3-dehydroshikimate dehydratase, a strategy that has been used successfully to engineer *Pseudomonas putida* KT2440 to generate intermediates on the β-ketoadipate pathway from d-glucose [8]. Via such an approach, engineered strains of *R. jostii* RHA1 could then potentially convert both lignin and cellulose fractions of lignocellulose into bio-products derived from protocatechuic acid.

Although rhodococci do not have the metabolic capability to degrade cellulose, engineering of *Rhodococcus opacus* PD630 to utilise cellobiose [9] and carboxymethylcellulose [10] as growth substrates has been previously reported by Hetzler et al. by expression of β-glucosidase genes *bglABC* [9] and *Cellulomonas fimi cenA* genes [10] respectively. Here we demonstrate the presence of beta-glucosidase enzymes in *R. jostii* RHA1, and test the expression of 6 Gram-positive cellulase genes, in order to permit utilisation of cellulose as a growth substrate. We also report the presence of a 3-dehydroshikimate dehydratase gene in *R. jostii* RHA1, needed to convert 3-dehydroshikimate to protocatechuic acid, and utilisation of quinic acid as growth substrate.

## 2 Materials and methods

**Materials.** Bacterial strains and plasmids used are described in Supporting Information Table S1. CenA from *Cellulomonas fimi* optimized for expression in *Rhodococcus* sp was synthesized from Genscript. Cel5A full length and only catalytic domain (Cel5AC) from *Bacillus subtilis*, Cel5AC.D, a chimeric enzyme comprising of catalytic domain of *Bacillus subtilis* Cel5A and *Caldicellulosiruptor besci* CelD were codon optimized and synthesized for expression in *Rhodococcus* from Fisher Scientific. Genes encoding Cel6A and Cel48 were amplified from genomic DNA extracted from *Thermobifida fusca yx*. Oligonucleotide primers for PCR amplification are listed in Supporting Information Table S2. DNA and protein sequences of expressed cellulase enzymes are detailed in Supporting Information Table S3. Carboxymethyl cellulose (CMC), 5x M9 salts, potassium sodium tartrate and dinitrosalicylic acid were purchased from Sigma Aldrich. Cellobiose was purchased from Argos Scientific.

### 2.1 Cloning into pTip vectors

PCR amplified/synthetic genes were restricted using Fast digest enzymes from Thermofisher Scientific. CenA was amplified from the synthetic construct to add restriction enzyme sites of *Nde*I and *Hind*III for cloning into pTipQC2 vector. T4 DNA ligase from NEB was used for ligation of the restricted genes into thiostrepton inducible expression vector pTipQC2 (vector map shown in Supporting Information Fig. S1) [11]. The ligation mix was kept overnight at 4 °C and then transformed into chemically competent *E. coli* Top10 cells. Ampicillin (100 μg/ml) was used as selection marker. Colony PCR was performed for the identification of transformed colonies containing recombinant plasmids (shown in Supporting Information Fig. S2). The confirmed colonies were inoculated into Luria-Bertani (LB) broth containing 100 μg/ml ampicillin and plasmids were extracted using NEB Plasmid extraction kit according to manufacturer's protocol. Double digestion and sequencing was used for insert confirmation.

### 2.2 Electroporation into competent *Rhodococcus jostii* RHA1 cells

The RHA1 cells were made electrocompetent by inoculating 1 ml refreshed cells in 50 ml LB at 30 °C with 180 rpm shaking till O. D_600_ reached 0.8 to 1. Cells were harvested using centrifugation (4000 rpm) at 4 °C. Cell pellets were washed three times with sterile 10% chilled glycerol after the final wash the cells were resuspended in 2.5 mls of 10% glycerol. Aliquots of 200 μl were made and stored at −80 °C till further use. 100 ng-1 μg of plasmid DNA pTipCenA, pTipCel6A, pTipCel5A, pTipCel5AC, pTipCel5AC.D and pTipCel48 A were mixed with the electrocompetent cells and kept on ice for 1 h. The samples were transferred to 2 mm electroporation cuvettes and electroporated using following condition: 2.5 kV, 25 μF and 400 Ω 1 ml of ice cold LB was added immediately following electroporation and placed at 30 °C overnight. Next morning the cells were centrifuged and cell pellets were concentrated to 200 μl and spread on LB Agar plates containing chloramphenicol (50 μg/ml). Colonies were visible after 2–3 days of incubation at 30 °C. Colony PCR was used for the identification of the transformed colonies.

### 2.3 Growth on cellulose substrates

Recombinant strains containing plasmids with cellulase genes were cultured on M9 agar containing 0.5% CMC, CaCl_2_, MgSO_4_ and trace elements at 30 °C. 1–2 μl of thiostrepton (10 mg/ml) was added on top of the colonies after 24 h. After 3 days the plates were stained with 0.1% Congo red solution [12] for 30 min and then washed three times with 1 M NaCl solution. The clear zones indicated the presence of cellulase activity (shown in Supporting Information Fig. S3) [12,13].

### 2.4 Quantitative assay for cellulase activity

A single colony of recombinant strains showing CMCase activities was used to inoculate 10 ml of sterile LB broth containing 50 μg/ml chloroamphenicol for 24 h, 1 ml of the culture was harvested by centrifugation at 6000 rpm and 4 °C, supernatant was discarded and the cell pellet was washed 3 times with sterile M9 salts, the washed pellet was used as inoculum for the cellulase producing M9 liquid media containing 0.5% CMC, CaCl_2_, MgSO_4_, trace elements and 50 μg/ml chloroamphenicol at 30 °C. Cultures were induced by addition of 5 μg/ml thiostrepton after 24 h. Aliquots were removed for measurement of OD_600_. Triplicate biological replicates were carried out, and the standard deviation of OD_600_ values determined. Cultures were also grown on the same media containing 0.1% glucose.

After 4 days 1 ml of the culture was harvested by centrifugation; 125 μl of supernatant was mixed with 125 μl of phosphate buffer pH 7 and 250 μl of 1% CMC solution was added as substrate and incubated at 30 °C for 20–30 min with shaking in thermomixer (Eppendorf). Total reducing sugars were measured using DNS assay [14]. 1 unit of enzyme activity was defined as the amount of enzyme required to produce 1 μmol reducing sugars per min under assay conditions. Wild type RHA1 and RHA1 containing empty pTip vector was also cultured in M9 medium with 0.5% CMC, CaCl_2_, MgSO_4_, trace elements and 0.1% glucose to check the background endoglucanase activity.

### 2.5 Cellobiose utilisation by wild type *R. jostii* RHA1

*R. jostii* RHA1 wild type cells were grown on M9 minimal media containing 1% cellobiose. 1 ml of overnight grown culture of RHA1 was harvested and washed three times with M9 salts and inoculated into M9 media containing 1% cellobiose and kept at 30 °C and 180 rpm shaking. The reducing sugars were measured using DNS assay after every 24 h in order to determine the consumption of cellobiose.

### 2.6 Quinic acid utilisation pathway

1 ml of overnight grown culture of *R. jostii* RHA1 was harvested and washed three times with M9 salts and inoculated into M9 media containing 0.5% quinic acid and kept at 30 °C and 180 rpm shaking. OD_600_ was measured after every 24 h.

## 3 Results

### 3.1 Existence of β-glucosidase genes in *Rhodococcus jostii* RHA1

The microbial conversion of cellulose into glucose requires three different types of glycosyl hydrolases: an endoglucanase that can hydrolyse the glucose-β-1,4-glucose within a cellulose chain to form β-glucose oligomers; an exoglucanase that can cleave β-glucose oligomers sequentially from the end of the glycan chain to form a cellobiose dimer; and β-glucosidase that can hydrolyse cellobiose to glucose. In their studies on *Rhodococcus opacus* PD630, Hetzler & Steinbüchel reported that this microbe lacked β-glucosidase genes, hence they imported exogenous β-glucosidase genes to improve its ability to utilise cellobiose [9]. They further episomally expressed endoglucanase and exoglucanase genes for the saccharification of cellulose [10]. We therefore first examined the genome of *Rhodococcus jostii* RHA1 for these three types of gene.

Bioinformatic analysis of the *R. jostii* RHA1 genome using the Uniprot database revealed no candidate endoglucanase or exoglucanase genes, however, there were two candidate β-glucosidase genes. RHA1_ro01034 encodes a 425 amino acid BglA homologue, which after database search using the BLAST algorithm revealed >60% sequence identity to 24 microbial β-glucosidases, 11 in the Rhodococcus genus, including accession W8HGX8 in *Rhodococcus opacus* PD630 (94.4% identity), listed in Supporting Information Fig. S4. BglA has been identified in *Clostridium thermocellulum* [15], *Bacillus polymyxa* [16], and several other cellulose-degrading bacteria [17], and is known to have catalytic activity for hydrolysis of cellobiose and larger oligosaccharides [16,17].

The second candidate was RHA1_ro02947, encoding a 759 amino acid β-glucosidase. Database searches using the BLAST algorithm revealed sequence similarity with β-glucosidases from a wider range of bacteria, of which the most abundant was the Microbacterium genus, and which contained only a small number of related *Rhodococcus* sequences (data listed in Supporting Information Fig. S4). Several related sequences are in the β-glucosidase B family, commonly referred to as BglB [17]. Bioinformatic analysis therefore suggests that *Rhodococcus jostii* RHA1 possesses both BglA and BglB β-glucosidase genes.

**Fig. 1:**
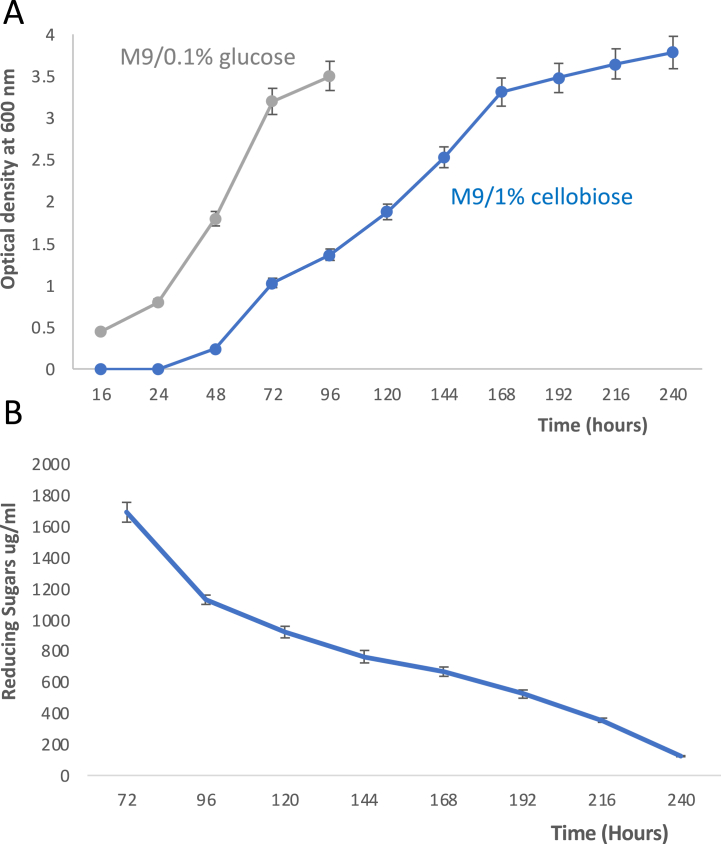
A. Growth of *Rhodococcus jostii* RHA1 on M9 minimal media containing either 1% cellobiose (blue) or 0.1% glucose (gray), measured by OD_600_. B. Reducing sugars measured during growth in M9/1% cellobiose versus incubation time, measured by reducing sugar assay. Error bars indicate standard deviation of triplicate biological replicates.

*R. jostii* RHA1 was then tested for its ability to utilise cellobiose as growth substrate. Growth was gradually observed at 30 °C on minimal M9 media containing 1% cellobiose, as shown in Fig. 1A. After a period of little or no growth for 48 h, A_600_ reached 1.0 after 72 h, and increased to 3.5 after 168 h. Aliquots were removed for assay of reducing sugars using the DNS assay [14], as shown in Fig. 1B, which showed >50% consumption of reducing sugars after 168 h, consistent with utilisation of cellobiose.

### 3.2 Expression of cellulase genes in *Rhodococcus jostii* RHA1

**Fig. 2:**
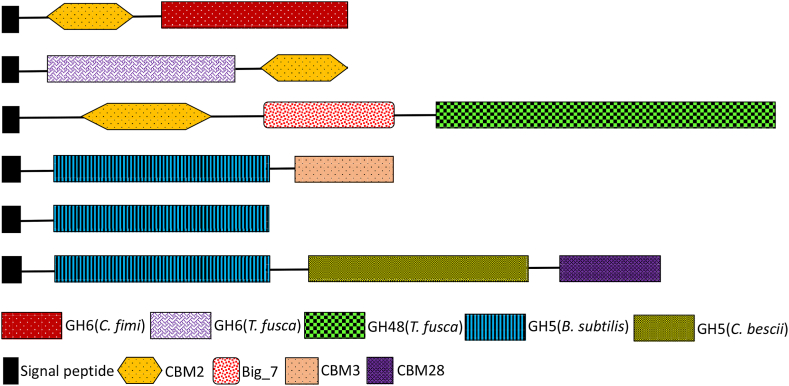
Maps of recombinant cellulase genes used, showing catalytic domains, signal peptides, and carbohydrate binding modules (CBMs) present in the cellulase genes. Sequences and signal peptides are given in Supporting Information Table 3.

Since *R. jostii* RHA1 was found to already utilise cellobiose for growth, in order to enable growth on cellulose, it was necessary to express endoglucanase and/or exoglucanase genes. Six cellulase genes were selected: endoglucanase *Cellulomonas fimi cenA* [18], which had been previously expressed in *R. opacus* PD630 [10]; endoglucanase *Thermobifida fusca cel6A* [19]; exoglucanase *Thermobifida fusca cel48A* [20]; endoglucanase *Bacillus subtilis cel5A* [21]; *cel5AC* (catalytic domain only of *Bscel5A*); and *cel5AC.D*, encoding a chimeric enzyme with the catalytic domain of *Bscel5A* and *Caldicellulosiruptor bescii celD* [22]. Each gene was cloned into expression vector pTipQC2, and transformed into *R. jostii* RHA1 via electroporation. The six cellulase constructs are illustrated in Fig. 2.

Endoglucanase activity was first assessed by growth on agar plates containing 0.5% CMC, and staining with Congo Red [13]. *R. jostii* pTipQC2-cenA, *R. jostii* pTipQC2-cel6A, *R. jostii* pTipQC2-cel5A, *R. jostii* pTipQC2-cel5AC and *R. jostii* pTipQC2-cel5AC.D showed a significant zone of clearing around the bacterial colonies, consistent with expression of endoglucanase activity, whereas no clear zone was observed with wild-type *R. jostii* RHA1, or with *R. jostii* pTipQC2 empty vector (see Supporting Information). Endoglucanase activity was then assessed quantitatively by DNS assay (see Table 1), which indicated that expression of *C. fimi* CenA gave the highest endoglucanase activity.

**Table 1:** Endoglucanase activities of the recombinant *R. jostii* strains expressing cellulase genes on pTipQC2 vector, measured using DNS assay. Errors were measured from standard deviation of triplicate assay readings. Activity for wild-type *R. jostii* RHA1 and *R. jostii* pTipQC2 was not detectable (<0.001 U/mL). ^a^Chimeric gene containing catalytic domain of *Bscel5A* and *Caldicellulosiruptor bescii celD*.

Cellulase gene expressed	Enzyme Activity (U/mL)
*C. fimi cenA*	0.209 ± 0.005
*B. subtilis cel5A*	0.153 ± 0.004
*T. fusca cel6A*	0.072 ± 0.004
*B. subtilis cel5AC*	0.147 ± 0.005
*cel5AC.D*^*a*^	0.155 ± 0.003

**Fig. 3:**
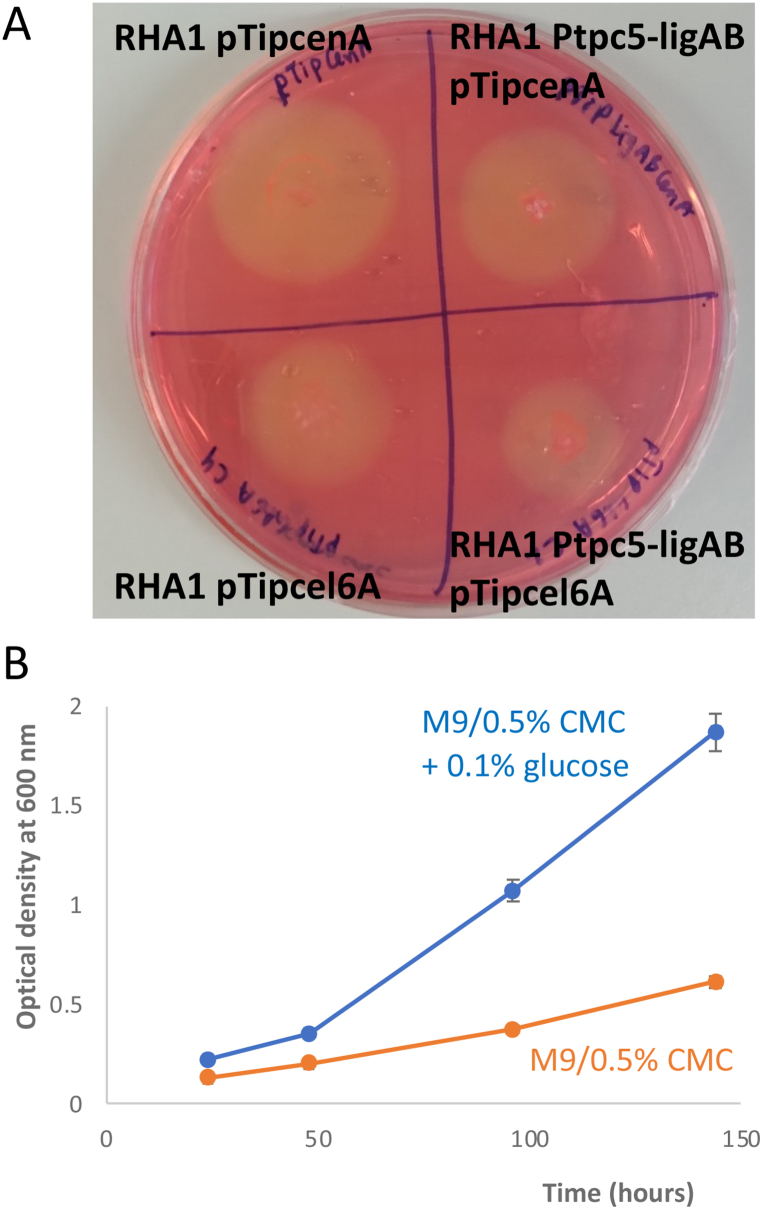
Growth of recombinant *R. jostii* strains on media containing carboxymethylcellulose (CMC). A. Growth of *R. jostii* pTipQC2-cenA and *R. jostii* pTipQC2-cel6A on agar containing 0.5% CMC, and staining with Congo Red (zone of clearance indicates cellulase activity). See Supporting Information Fig. S3 for growth of other recombinant *R. jostii* strains, and lack of growth by wild-type *R. jostii* RHA1. B. Growth of *R. jostii* pTipQC2-cel6A in liquid M9 media containing 0.5% CMC (orange), and 0.5% CMC + 0.1% glucose (blue). No growth observed for wild-type *R. jostii* RHA1 on M9/0.5% CMC. Error bars indicate standard deviation of triplicate biological replicates.

The ability to utilise carboxymethylcellulose (CMC) as growth substrate was then examined. Growth of recombinant *R. jostii* strains was observed on M9/0.5% CMC agar plates (shown for *R. jostii* pTipQC2-cenA and *R. jostii* pTipQC2-cel6A in Fig. 3A), together with a zone of clearance upon staining with Congo Red, however, no growth was observed for wild-type *R. jostii* RHA1 (see Supporting Information Fig. S3). Growth of recombinant *R. jostii* strains was also observed in liquid M9 media containing 0.5% carboxymethylcellulose (CMC), which is shown for *R. jostii* pTipQC2-cel6A in Fig. 3B, reaching OD_600_ 0.61 after 144 h. Growth in M9 media containing 0.5% CMC and 0.1% glucose reached OD_600_ 1.1 after 72 h (see Fig. 3B).

Aliquots from the culture supernatant for *R. jostii* pTipQC2-cenA grown on M9 containing 0.5% CMC/0.1% glucose were assayed for production of reducing sugars from CM cellulose via DNS assay, giving an activity of 0.2 U/ml after 4 days. Wild type *R. jostii* RHA1 and the strain containing empty pTip vector were also grown in the same way with 0.1% glucose, but no endoglucanase activity was observed in the culture supernatant.

### 3.3 Co-culture of *R. jostii* pTipQC2-cenA and *R. jostii* pTipQC2-cel5A with *R. jostii* pTipQC2-cel48

**Fig. 4:**
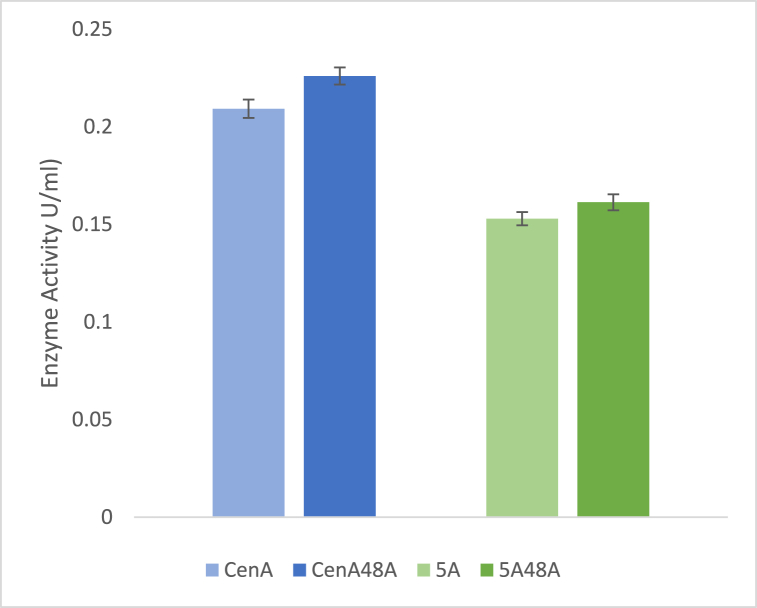
Extracellular cellulase activities of co-cultures of *R. jostii* pTipQC2-cenA (endoglucanase) and *R. jostii* pTipQC2-cel5A (endoglucanase) with *R. jostii* pTipQC2-cel48 A (exoglucanase), measured by DNS assay. Co-culture activities in dark blue (CenA-Cel48 A) and dark green (Cel5A-Cel48 A). Error bars indicate standard deviation of triplicate assay readings.

In order to examine whether the presence of both endocellulase and exocellulase would provide more efficient utilisation of cellulose, *R. jostii* strains producing CenA or Cel5A (endoglucanase) were co-cultured with the Cel48 A (exoglucanase) producing strain*.* Aliquots were taken after 4 days of incubation and were used to check the CMCase activity. DNS assay was carried out to determine the amount of reducing sugars. As shown in Fig. 4, there was a slight increase in the amount of reducing sugars produced, an 8% increase in the case of CenA, and a 6% increase in the case of Cel5A. In both cases the increase was statistically significant, but was a modest effect, implying that there is not a large benefit in expressing both extracellular cellulase enzymes.

### 3.4 Existence of 3-dehydroshikimate dehydratase in *Rhodococcus jostii* RHA1

Having established the ability of engineered *R. jostii* strains to convert carboxymethylcellulose into reducing sugars, we then investigated the possibility of converting d-glucose into aromatic products, via the shikimate pathway, used by bacteria to biosynthesise l-phenylalanine, l-tyrosine and l-tryptophan from glucose. Johnson et al. have engineered *Pseudomonas putida* KT2440 to generate products from the β-ketoadipate pathway from d-glucose as carbon source, by insertion of the *asbF* gene encoding 3-dehydroshikimate dehydratase, which converts pathway intermediate 3-dehydroshikimic acid into protocatechuic acid [8].

Bioinformatic analysis using the Uniprot database revealed that, remarkably, *R. jostii* RHA1 contains a gene predicted to encode 3-dehydroshikimate dehydratase, RHA1_ro01367. Adjacent to this gene on the genome is a 3-dehydroquinate dehydratase gene RHA1_ro01368, but no other shikimate pathway genes are located nearby, and there is a second 3-dehydroquinate dehydratase gene RHA1_ro03051 located elsewhere on the genome. The function of ro01367 and ro01368 may relate to utilisation of quinic acid, a compound found in many plants, since several bacteria are known to metabolise quinic acid [[23], [24], [25]], including *Rhodococcus rhodocrous* N75 [24]. We therefore hypothesised that genes ro01367 and ro01368 might be utilised by *R. jostii* RHA1 for catabolism of quinic acid.

**Fig. 5:**
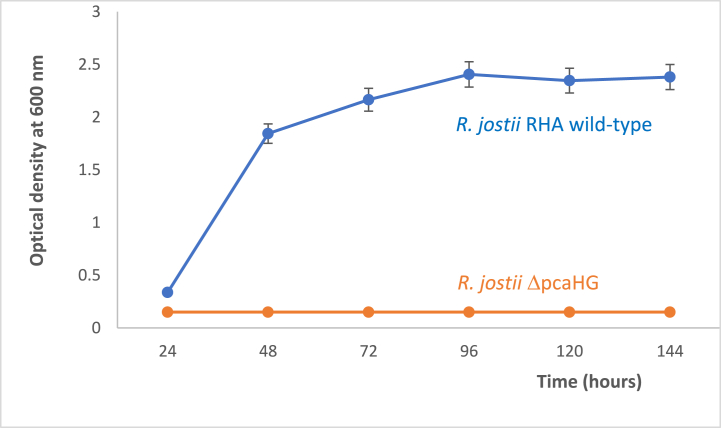
Growth of wild-type *R. jostii* RHA1 on M9/0.5% quinic acid (in blue), compared with growth of *R. jostii* ΔpcaHG (in orange). Error bars indicate standard deviation of triplicate biological replicates.

The ability of *R. jostii* RHA1 to utilise quinic acid for growth was tested by growth in minimal M9 media containing 0.5% quinic acid at 30 °C. As shown in Fig. 5, strong growth was observed, reaching A_600_ 2.4 after 96 h. Holder et al. have also tested both *R. opacus* PD630 and *R. jostii* RHA1 for growth on a range of carbon sources, and their data also qualitatively indicated growth of both strains on quinic acid as growth substrate [26].

**Fig. 6:**
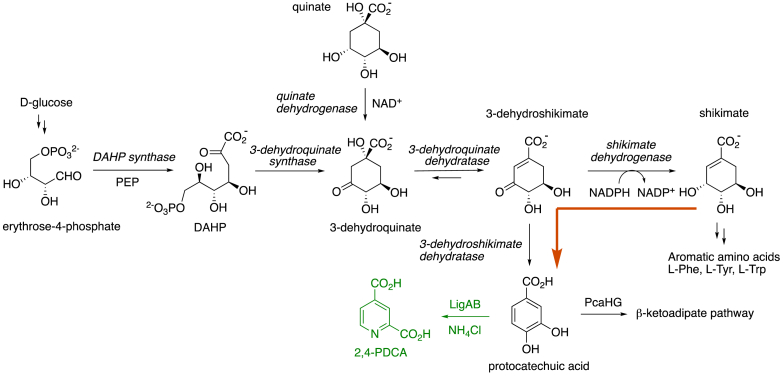
Biochemical steps of the shikimate pathway, showing the reactions catalysed by quinate dehydrogenase and 3-dehydroshikimate dehydratase. Formation of bioproduct 2,4-PDCA via LigAB biotransformation is shown in green. The formation of protocatechuic acid from shikimate is shown in orange.

In order to investigate this catabolic pathway further, we tested a ΔpcaHG gene knockout generated previously in *R. jostii* RHA1, in which the *pcaHG* genes encoding protocatechuate 3,4-dioxygenase, the first step on the β-ketoadipate pathway, have been deleted [6]. The *R. jostii* ΔpcaHG knockout strain showed no growth on M9/quinic acid media (see Fig. 5), implying that the β-ketoadipate pathway is required for growth on quinic acid. This observation is consistent with the expected metabolism of protocatechuic acid via the β-ketoadipate pathway to the citric acid cycle (see Fig. 6).

Utilisation of quinic acid by bacteria involves the initial oxidation of quinic acid to 3-dehydroquinic acid, as shown in Fig. 6, which is catalysed in *Rhodococcus rhodocrous* N75 by a 44 kDa bifunctional quinate/shikimate dehydrogenase enzyme [24]. There is no annotated quinate dehydrogenase gene on the *R. jostii* RHA1 genome, however, there are four *aroE* genes that are annotated to encode shikimate dehydrogenase. Since quinic acid and shikimic acid have related chemical structures, and some enzymes are reported to possess activity for both shikimic acid and quinic acid [24,27], it is feasible that a gene annotated as an *aroE* gene encodes a quinate dehydrogenase or bifunctional activity. The *aroE4* gene RHA1_ro07138 is located adjacent to the *aroBCK* genes encoding shikimate pathway enzymes (ro07142, ro07140, ro07141 respectively), consistent with this gene being used for aromatic amino acid biosynthesis. However, the *aroE1* gene RHA1_ro01342 is located immediately adjacent to the *pcaHGBLRF* gene cluster (ro01335-ro01340) encoding β-ketoadipate pathway enzymes, so we hypothesise that the *aroE1* gene product may be used as a catabolic quinate dehydrogenase enzyme activity for quinic acid utilisation. The remaining *aroE* genes are RHA1_ro01564 (*aroE2*) and RHA1_ro01853 (*aroE3*), which are neither co-located with shikimate pathway genes, nor with aromatic degradation genes.

In order to investigate whether the 3-dehydroshikimate dehydratase gene present in *R. jostii* RHA1 could readily generate an aromatic bioproduct from cellulose substrates, pTipQC2-cenA was transformed into a *R. jostii* pcaHGligAB strain generated previously, in which the *pcaHG* genes have been replaced by *Sphingobium* SYK-6 *ligAB* genes encoding protocatechuate 4,5-dioxygenase, which generates a bioproduct 2,4-pyridine-dicarboxylic acid (2,4-PDCA) from lignin degradation, shown in Fig. 6 [6]. *R. jostii* pcaHGligAB pTipQC2-cenA was grown on minimal M9 media containing 0.5% CM cellulose for 7 days, and culture supernatant analyzed by HPLC, but no 2,4-PDCA was observed.

**Fig. 7:**
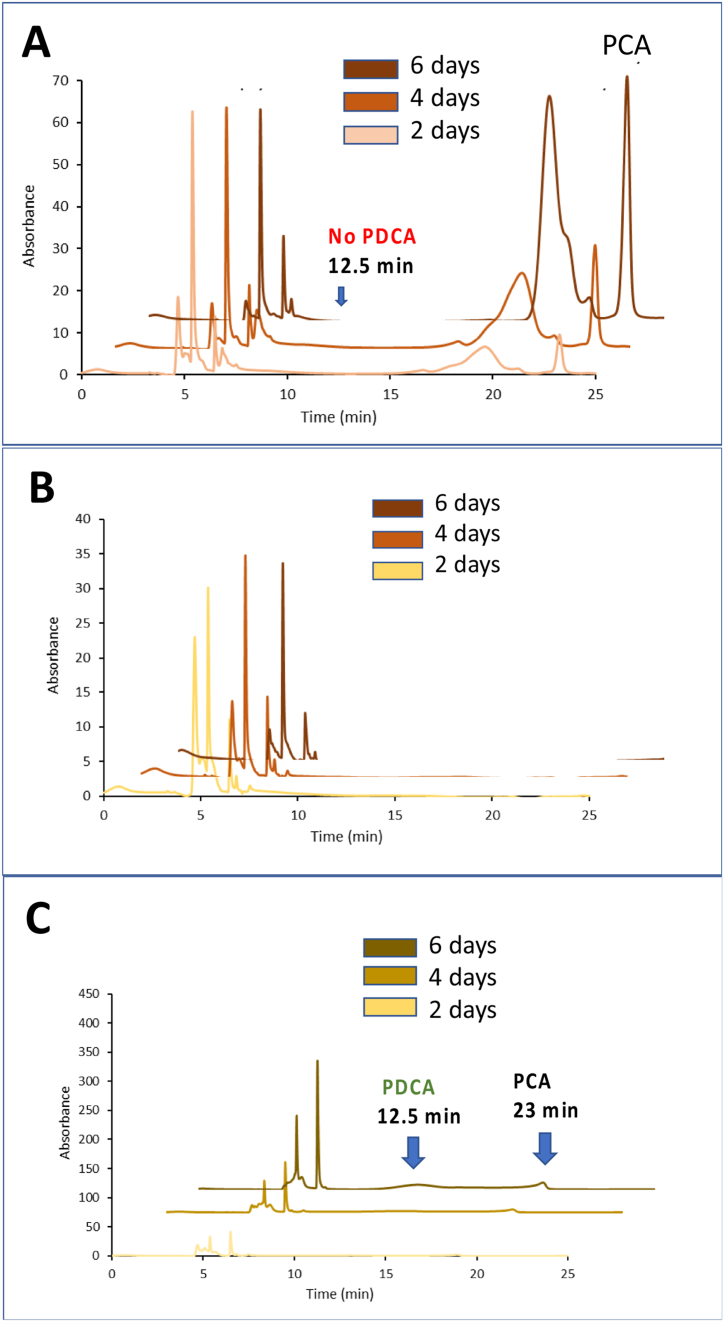
Conversion of different carbon sources to protocatechuic acid (PCA) and 2,4-pyridine-dicarboxylic acid (PDCA) by *R. jostii* pcaHGligAB containing an empty pTipQC2 vector. A. M9 media containing 0.1% shikimic acid and 0.1% glucose, showing formation of PCA but not PDCA. B. M9 media containing 0.1% d-glucose. C. M9 media containing 0.1% glycerol, showing formation of PCA and 2,4-PDCA.

To explore possible reasons for this observation, *R. jostii* pcaHGligAB containing an empty pTipQC2 vector was further tested for conversion of different carbon sources to protocatechuic acid (PCA) and 2,4-PDCA. Using 0.1% shikimic acid in the presence of 0.1% glucose, conversion to PCA was observed (see Fig. 7A, concentration 7.5 mg/L), but not 2,4-PDCA. Using M9 containing 0.1% glucose, no aromatic products were observed (see Fig. 7B). Using M9 containing 0.1% glycerol as growth substrate, small amounts of PCA (concentration 4 mg/L) and 2,4-PDCA (concentration 3 mg/L) were observed (see Fig. 7C). These data show that it is possible to generate PCA and 2,4-PDCA from the shikimate pathway, but that further metabolic engineering will be needed for efficient conversion, as discussed in the Discussion.

## 4 Discussion

The work described here demonstrates that *R. jostii* RHA1 can be engineered to grow on carboxymethylcellulose as growth substrate, and that efficient conversion to reducing sugars is observed (see Fig. 3). Of the cellulase genes tested, *Cellulomonas fimi* CenA appears to function most efficiently in *Rhodococcus jostii* RHA1, and this enzyme was found to be effective in *Rhodococcus opacus* PD630 [10]. Wild-type *R. jostii* RHA1 can grow on cellobiose as growth substrate, which can be rationalised by the presence of *bglA* and *bglB* β-glucosidase genes in its genome. We note that *R. opacus* PD360 contains a *bglA* gene but not a *bglB* gene, and this strain was reported not to grow on cellobiose as growth substrate [9], therefore, the presence of a *bglB* gene, or the combination of these two genes, appears significant.

Furthermore, *R. jostii* RHA1 contains an active 3-dehydroshikimate dehydratase gene, which converts 3-dehydroshikimic acid to protocatechuic acid, and which rationalises the observed growth on quinic acid as growth substrate (see Fig. 5). We note that quinic acid is often found as a conjugate with caffeic acid, chlorogenic acid [28], and since *R. jostii* RHA1 is a highly efficient aromatic degrader, it is probable that *R. jostii* RHA1 can degrade both quinic acid and caffeic acid components of chlorogenic acid.

Although *R. jostii* pcaHGligAB pTipQC2-cenA was not able to generate 2,4-PDCA from carboxymethylcellulose as growth substrate, there are several possible explanations for why 2,4-PDCA was not produced in this initial experiment. 1. The shikimate pathway is known to be subject to feedback inhibition of the first enzyme DAHP synthase, however, the molecular basis for this feedback regulation is understood, and can be alleviated through genetic modification [29,30]. 2. Catabolite repression of aromatic degradation pathways is observed in *R. jostii* RHA1 in the presence of d-glucose [31]. The observation that neither PCA nor 2,4-PDCA were formed by *R. jostii* pcaHGligAB in media containing d-glucose (see Fig. 7B) is consistent with catabolite repression by d-glucose. 3. *R. jostii* RHA1 channels flux from d-glucose into lipid biosynthesis under low nitrogen conditions [26,32]. The formation of small amounts of PCA and 2,4-PDCA by *R. jostii* pcaHGligAB when grown on glycerol as carbon source (see Fig. 7C) shows that it is possible to use the native 3-dehydroshikimate dehydratase to generate PCA and 2,4-PDCA. However, the titres of 2,4-PDCA observed here are significantly lower than those observed from lignin degradation upon expression of *Sphingobium* SYK-6 *ligAB* in wild-type *R. jostii* RHA1 (100–125 μg/mL) [5] or in a further engineered strain (240–330 μg/mL) [6]. Hence, in order to efficiently channel flux from cellulose breakdown into formation of protocatechuic acid, further metabolic engineering will be required. Nevertheless, the ability to convert cellulose to reducing sugars, and to convert quinic acid to protocatechuic acid, makes *R. jostii* RHA1 a promising microbe for further engineering approaches to convert both lignin and cellulose into protocatechuic acid-derived products.

## 5 Conclusion

This work demonstrates that *Rhodococcus jostii* RHA1 can be engineered to utilise carboxymethylcellulose as growth substrate, by plasmid-based insertion of endoglucananase genes. *R. jostii* RHA1 also contains a native 3-dehydroshikimate dehydratase gene, which provides a metabolic route to convert 3-dehydroshikimate to protocatechuic acid (PCA). The ability to utilise both cellulose and lignin components of lignocellulose, and the metabolic ability to convert both cellulose and lignin into PCA, offers potential for engineering of this microbe to generate higher titres of PCA-derived bioproducts from plant biomass feedstocks.

## Author contribution statement

Conceived and designed the experiments: Imran Ali, Timothy D.H. Bugg. Performed the experiments: Rabia Yasin, Goran M.M. Rashid. Analyzed and interpreted the data: Rabia Yasin, Goran M.M. Rashid, Imran Ali, Timothy D.H. Bugg. Wrote the paper: Rabia Yasin, Timothy D.H. Bugg.

## Funding statement

Professor Timothy Bugg and Dr Goran Rashid were supported by the 10.13039/501100000268Biotechnology and Biological Sciences Research Council {BB/T010622/1}.

Rabia Yasin was supported by the Higher Education Commision of Pakistan International Research Support Initiative Program. She was awarded a scholarship for a 6 month visit to the University of Warwick.

### Data availability statement

Data included in article/supp. Material/referenced in article.

## Declaration of competing interest

The authors declare that they have no known competing financial interests or personal relationships that could have appeared to influence the work reported in this paper.
