# Supplementary material for: Engineering of Rhodococcus jostii RHA1 for utilisation of carboxymethylcellulose
Source: Heliyon. 2023 Aug 25;9(9):e19511. doi: 10.1016/j.heliyon.2023.e19511 (PMC10558750; doi:10.1016/j.heliyon.2023.e19511)
Supplement: Multimedia component 1 [file mmc1.pdf]

Engineering of *Rhodococcus jostii* RHA1 for utilisation of carboxymethylcellulose

Rabia Yasin, Goran M.M. Rashid, Imran Ali, and Timothy D.H. Bugg*

Supporting Information

Table S1. Bacterial strains and plasmids used

Table S2. List of Oligonucleotide Primers used

Table S3. Gene/protein sequences for expression in *R. jostii* RHA1

Table S4 BLAST results for *R. jostii* RHA1 beta-glucosidase genes

Table S5. List of chromosomal *R. jostii* RHA1 genes referred to in this study

Figure S1. Vector Map for pTipQC2

Figure S2. Agarose Gels for PCR Amplification, digests and colony PCR.

Figure S3. Congo Red Staining of *R. jostii* pTipQC2-cellulase constructs, grown on M9 agar containing (A) 0.5% CMC + 0.1% glucose (B) 0.5% CMC (without glucose).

Table S1. Bacterial strains and plasmids used:

**Strains or Plasmids**	**Characteristics**
**Strains:**	
E. coli Top 10	F- mcrA Δ(mrr-hsdRMS-mcrBC) φ80lacZΔM15 ΔlacX74 nupG recA1 araD139 Δ(ara-leu)7697 galE15 galK16 rpsL(Str^R) endA1 λ^-
R.jostii RHA1	
R.jostii pcaHG::ligAB	replacement of *pcaHG* genes by *ligAB* (constitutive Ptpc5 promoter)
ΔpcaHG RHA1	*ΔpcaHG* gene deletion strain
Plasmids:	
PTipQC2-cenA	cenA as Nde1-HindIII fragment
PTipQC2-cel6A	cel6A as NdeI-XhoI fragment
PTIPQC2-cel5A	cel5A as NdeI-HindIII fragment
PTipQC2-cel48A	cel48A as NdeI-XhoI fragment
PTipQC2-cel5AC	cel5AC as NdeI-HindIII fragment
PtipQC2-cel5AC.D	cel5AC.D as NdeI-Xho1 Fragment

Table S2. List of Oligonucleotide Primers used

	**Forward Primer 5' to 3'**	**Reverse Primer 5' to 3'**
**CenA**	AGAGAA**CATATG**AGCACCCGGCGG	CCAGATCT**AAGCTT**TCACCAGCGGGCG
**Cel6A**	GCC C**CA TAT G**TC CCC CAG ACC TC	CAC TGC **CTC GAG** GGG TCA GCT G
**Cel48A**	CAA **CAT ATG** AGA TCG TTA CTG TCT CCC CG	TCC GGC **CTC GAG** CAG TTC GTC GTG
**pTip**	GGCTTGCACCTCACGTCA	CTCCGTGTGTTTGTGCAGGT

Table S3. Gene/protein sequences for expression in *R. jostii RHA1*

CenA from *Cellulomonas fimi* Optimized Sequence Length:1368, GC%:72.91

ATGTCGACCCGGCGGACGGCGGCGGCGCTCCTGGCGGCGGCGGTGGCGGTGGGCGGC
CTCACGGCGCTGACGACGACGGCGGCGCAGGCGGCCCCGGGCTGCCGCGTGGACTACGCC
GTGACCAACCAGTGGCCGGGCGGCTTCGGCGCGAACGTCACCATCACCAACCTGGGCGAC
CCGGTGTCGTCGTGGAAGCTCGACTGGACCTACACCGCGGGCCAGCGCATCCAGCAGCTG
TGGAACGGCACCGCCTCCACCAACGGTGGCCAGGTGTCGGTCACCTCGCTCCCGTGGAAC
GGCTCCATCCCGACCGGCGGCACCGCCTCGTTCGGCTTCAACGGCTCCTGGGCGGGCTCC
AACCCGACCCCGGCGTCGTTCTCCCTCAACGGCACCACCTGCACCGGCACCGTCCCGACC
ACCTCGCCGACCCCGACCCCGACCCCGACGACCCCGACCCCGACCCCGACCCCGACCCCG
ACCCCGACCCCGACAGTCACCCCGCAGCCGACCTCGGGCTTCTACGTGGACCCGACCACC
CAGGGCTACCGCGCCTGGCAGGCGGCCTCCGGCACCGACAAGGCGCTGCTCGAGAAGATC
GCCCTGACCCCGCAGGCGTACTGGGTGGGCAACTGGGCGGACGCGTCGCACGCCCAGGCC
GAGGTCGCCGACTACACCGGCCGCGCCGTGGCGGCCGGCAAGACCCCGATGCTCGTCGTG
TACGCCATCCCGGGCCGCGACTGCGGCTCGCACTCGGGCGGCGGCGTCTCGGAGTCGGAG
TACGCCCGCTGGGTGGACACCGTGGCCCAGGGCATCAAGGGCAACCCGATCGTGATCCTC
GAGCCGGACGCCCTGGCCCAGCTCGGCGACTGCTCGGGCCAGGGCGACCGCGTCGGCTTC
CTCAAGTACGCGGCCAAGTCGCTGACCCTCAAGGGCGCCCGCGTGTACATCGACGCGGGC
CACGCCAAGTGGCTGTCGGTCGACACCCCGGTGAACCGCCTCAACCAGGTCGGCTTCGAG
TACGCCGTGGGCTTCGCGCTGAACACCTCGAACTACCAGACCACCGCGGACTCCAAGGCG
TACGGCCAGCAGATCTCCCAGCGCCTCGGCGGCAAGAAGTTCGTCATCGACACCTCGCGC
AACGGCAACGGCTCCAACGGCGAGTGGTGCAACCCGCGGGGCCGCGCCCTGGGCGAGCGC
CCGGTCGCGGTGAACGACGGCTCCGGCCTGGACGCCCTGCTCTGGGTGAAGCTCCCGGGC
GAGTCGGACGGCGCGTGCAACGGTGGCCCGGCCGCCGGCCAGTGGTGGCAGGAGATCGCC
CTCGAGATGGCCCGCAACGCCCGCTGGTGA

Protein Sequence (Tat signal peptide underlined)

<u>MSTRRTAAALLAAAAVAVGGLTALTTTAAQ</u>AAPGCRVDYAVTNQWPGGFGANVTITNLGD
PVSSWKLDWTYTAGQRIQQLWNGTASTNGGQVSVTSLPWNGSIPTGGTASFGFNGSWAGS
NPTPASFSLNGTTCTGTVPTTSPTPTPTPTTPTPTPTPTPTPTPTVTPQPTSGFYVDPTT
QGYRAWQAASGTDKALLEKIALTPQAYWVGNWADASHAQAEVADYTGRAVAAGKTPMLVV
YAIPGRDCGSHSGGGVSESEYARWVDTVAQGIKGNPIVILEPDALAQLGDCSGQGDRVGF
LKYAAKSLTLKGARVYIDAGHAKWLSVDTPVNRLNQVGFEYAVGFALNTSNYQTTADSKA
YGQQISQRLGGKKFVIDTSRNGNGSNGEWCNPRGRALGERPVAVNDGSGLDALLWVKLPG
ESDGACNGGPAAGQWWQEIALEMARNARW-

Theoretical pI/Mw: 7.56 / 46730.95

Cel5A from *Bacillus subtilis* Optimized Sequence, Length: 1506, GC%: 59.05%

ATGAAGCGGTCGATTTCGATTTTCATCACGTGCCTGCTCATCACCCTCCTCACGATGGGC
GGCATGATCGCGTCCCCGGCCAGCGCCGCCGGCACCAAGACGCCCGTGGCCAAGAACGGA
CAGTTGTCCATCAAGGGGACCCAGCTGGTGAACCGCGATGGGAAGGCCGTTCAGCTGAAG
GGCATCAGCAGCCACGGCCTTCAGTGGTACGGCGAATACGTCAACAAGGATTCACTGAAG
TGGCTCCGCGACGACTGGGGTATCACTGTATTCCGCGCCGCGATGTACACCGCCGACGGT
GGCTACATCGACAACCCGTCGGTGAAGAATAAGGTCAAGGAGGCGGTCGAGGCCGCGAAG
GAACTCGGAATCTACGTCATCATTGACTGGCACATCCTGAATGATGGCAACCCTAACCAG
AACAAAGAGAAGGCGAAGGAGTTCTTCAAGGAGATGAGCTCGCTGTACGGGAACACGCCG
AATGTCATCTACGAGATCGCGAACGAACCGAACGGCGACGTGAACTGGAAGCGAGACATC
AAGCCGTATGCCGAGGAGGTCATCTCGGTGATCCGCAAGAACGACCCGGACAACATCATC
ATCGTCGGGACGGGAACCTGGAGCCAGGACGTCAACGATGCGGCGGACGACCAGCTCAAG
GACGCCAACGTCATGTACGCGTTGCACTTCTACGCAGGTACCCACGGCCAGTTCCTCCGG
GACAAGGCGAACTACGCCCTGTCGAAGGGCGCGCCGATCTTCGTCACCGAATGGGGCACC
TCTGACGCCTCCGGCAACGGCGGTGTGTTCCTGGACCAGAGCCGTGAGTGGCTGAAGTAC
CTCGACTCCAAGACGATCTCGTGGGTCAACTGGAATCTCTCGGACAAGCAGGAGTCGTCG
TCCGCTCTAAAACCGGGAGCCAGTAAAACCGGCGGCTGGCGGCTGTCCGACCTGTCCGCG
TCGGGTACGTTTGTCAGGGAGAACATCCTCGGAACCAAAGACTCGACCAAGGACATCCCC
GAGACCCCGTCCAAAGACAAGCCCACGCAGGAAAACGGCATCAGTGTGCAGTACCGCGCG
GGCGACGGCTCGATGAACTCCAACCAAATCCGGCCGCAGCTGCAAATCAAGAACAACGGG
AACACCACCGTCGACCTCAAGGACGTGACCGCACGGTACTGGTACAAGGCGAAAAACAAG
GGGCAGAACTTCGACTGCGACTATGCGCAGATCGGATGCGGCAATGTGACACATAAGTTC
GTGACCCTGCACAAGCCCAAGCAGGGTGCCGACACCTACCTGGAACTCGGCTTCAAGAAC
GGTACCCTCGCACCCGGCGCGTCGACTGGGAACATCCAGCTGCGCTTACACAACGACGAT
TGGTCAAACTACGCCCAGTCCGGGGACTACAGCTTCTTCAAGTCCAACACGTTCAAGACG
ACGAAGAAGATCACCCTCTACGACCAGGGCAAGCTGATCTGGGGGACCGAGCCCAACGGA
TCC**TGA**

Protein Sequence (Sec signal peptide underlined)

MKRSISIFITCLLITLLTMGGMIASPASAAGTKTPVAKNGQLSIKGTQLVNRDGKAVQLK
GISSHGLQWYGEYVNKDSLKWLRDDWGITVFRAAMYTADGGYIDNPSVKNKVKEAVEAAK
ELGIYVIIDWHILNDGNPNQNKEKAKEFFKEMSSLYGNTPNVIYEIANEPNGDVNWKRDI
KPYAEEVISVIRKNDPDNIIIVGTGTWSQDVNDAADDQLKDANVMYALHFYAGTHGQFLR
DKANYALSKGAPIFVTEWGTSDASGNGGVFLDQSREWLKYLDSKTISWVNWNLSDKQESS
SALKPGASKTGGWRLSDLSASGTFVRENILGTKDSTKDIPETPSKDKPTQENGISVQYRA
GDGSMNSNQIRPQLQIKNNGNTTVDLKDVTARYWYKAKNKGQNFDCDYAQIGCGNVTHKF
VTLHKPKQGADTYLELGFKNGTLAPGASTGNIQLRLHNDDWSNYAQSGDYSFFKSNTFKT
TKKITLYDQGKLIWGTEPNGS-

Theoretical pI/Mw: 8.55 / 55431.12

Cel6A from *Thermobifida fusca* Original Sequence Length: 1332, GC%: 68.2%

ATGTCCCCCAGACCTCTTCGCGCTCTTCTGGGCGCCGCGGCGGCGGCCTTGGTCAGCGCG
GCTGCTCTGGCCTTCCCGTCGCAAGCGGCGGCCAATGATTCTCCGTTCTACGTCAACCCC
AACATGTCCTCCGCCGAATGGGTGCGGAACAACCCCAACGACCCGCGTACCCCGGTAATC
CGCGACCGGATCGCCAGCGTGCCGCAGGGCACCTGGTTCGCCCACCACAACCCCGGGCAG
ATCACCGGCCAGGTCGACGCGCTCATGAGCGCCGCCCAGGCCGCCGGCAAGATCCCGATC
CTGGTCGTGTACAACGCCCCGGGCCGCGACTGCGGCAACCACAGCAGCGGCGGCGCCCCC
AGTCACAGCGCCTACCGGTCCTGGATCGACGAATTCGCTGCCGGACTGAAGAACCGTCCC
GCCTACATCATCGTCGAACCGGACCTGATCTCGCTGATGTCGAGCTGCATGCAGCACGTC
CAGCAGGAAGTCCTGGAGACGATGGCGTACGCGGGCAAGGCCCTCAAGGCCGGGTCCTCG
CAGGCGCGGATCTACTTCGACGCCGGCCACTCCGCGTGGCACTCGCCCGCACAGATGGCT
TCCTGGCTCCAGCAGGCCGACATCTCCAACAGCGCGCACGGTATCGCCACCAACACCTCC
AACTACCGGTGGACCGCTGACGAGGTCGCCTACGCCAAGGCGGTGCTCTCGGCCATCGGC
AACCCGTCCCTGCGCGCGGTCATCGACACCAGCCGCAACGGCAACGGCCCCGCCGGTAAC
GAGTGGTGCGACCCCAGCGGACGCGCCATCGGCACGCCCAGCACCACCAACACCGGCGAC
CCGATGATCGACGCCTTCCTGTGGATCAAGCTGCCGGGTGAGGCCGACGGCTGCATCGCC
GGCGCCGGCCAGTTCGTCCCGCAGGCGGCCTACGAGATGGCGATCGCCGCGGGCGGCACC
AACCCCAACCCGAACCCCAACCCGACGCCCACCCCCACTCCGACCCCCACGCCGCCTCCC
GGCTCCTCGGGGGCGTGCACGGCGACGTACACGATCGCCAACGAGTGGAACGACGGCTTC
CAGGCGACCGTGACGGTCACCGCGAACCAGAACATCACCGGCTGGACCGTGACGTGGACC
TTCACCGACGGCCAGACCATCACCAACGCCTGGAACGCCGACGTGTCCACCAGCGGCTCC
TCGGTGACCGCGCGGAACGTCGGCCACAACGGAACGCTCTCCCAGGGAGCCTCCACAGAG
TTCGGCTTCGTCGGCTCTAAGGGCAACTCCAACTCTGTTCCGACCCTTGGATCCAAGCTT
AGATCTCTCGAGCATCACCATCACCATCAC**TGA**

Protein Sequence: (Sec signal peptide underlined)

MMSPRPLRALLGAAAAALVSAAALAFPSQAAANDSPFYVNPNMSSAEWVRNNPNDPRTPV
IRDRIASVPQGTWFAHHNPGQITGQVDALMSAAQAAGKIPILVVYNAPGRDCGNHSSGGA
PSHSAYRSWIDEFAAGLKNRPAYIIVEPDLISLMSSCMQHVQQEVLETMAYAGKALKAGS
SQARIYFDAGHSAWHSPAQMASWLQQADISNSAHGIATNTSNYRWTADEVAYAKAVLSAI
GNPSLRAVIDTSRNGNGPAGNEWCDPSGRAIGTPSTTNTGDPMIDAFLWIKLPGEADGCI
AGAGQFVPQAAYEMAIAAGGTNPNPNPNPTPTPTPTPTPPPGSSGACTATYTIANEWNDG
FQATVTVTANQNITGWTVTWTFTDGQTITNAWNADVSTSGSSVTARNVGHNGTLSQGAST
EFGFVGSKGNSNSVPTLGSKLRSLEHHHHHH-

Theoretical pI/Mw: 6.18 / 47235.32

Cel5AC.D Chimeric enzyme Optimized Sequence: Length: 2535, GC%: 58.2

ATGAAGCGCTCCATCTCGATCTTCATCACCTGCCTGCTGATCACGCTCCTCACGATGGGC
GGCATGATCGCGTCCCCCGCGAGCGCCGCCGGCACCAAGACTCCGGTGGCGAAAAACGGG
CAGTTGAGCATCAAGGGCACACAGCTAGTGAACCGCGACGGCAAGGCGGTGCAACTGAAG
GGTATCTCGTCCCACGGGCTCCAGTGGTACGGAGAGTACGTAAATAAGGATAGCCTGAAG
TGGCTCCGTGATGATTGGGGTATCACCGTCTTCCGCGCAGCGATGTACACCGCCGACGGA
GGATACATTGATAATCCCTCGGTCAAGAACAAGGTCAAGGAGGCCGTCGAAGCCGCGAAG
GAATTGGGCATCTACGTCATCATCGACTGGCACATCCTCAACGACGGGAACCCGAACCAG
AACAAAGAGAAGGCCAAGGAGTTCTTCAAGGAGATGAGCTCCCTCTACGGCAACACCCCC
AACGTTATCTACGAGATCGCCAACGAGCCCAATGGTGACGTGAATTGGAAGCGAGACATC
AAGCCGTACGCCGAGGAGGTCATCTCCGTCATCCGCAAGAATGACCCGGACAACATCATC
ATCGTGGGCACTGGCACGTGGTCCCAGGACGTCAACGACGCCGCGGACGACCAGCTCAAA
GACGCGAACGTCATGTACGCCCTGCACTTCTACGCCGGAACGCACGGCCAGTTTCTTCGG
GACAAGGCCAACTACGCCCTGTCGAAGGGCGCCCCCATCTTCGTCACCGAGTGGGGAACC
AGTGATGCGAGCGGAAATGGCGGTGTCTTCCTCGACCAGTCGCGGGAATGGCTGAAGTAT
CTCGACAGCAAGACGATCTCGTGGGTCAACTGGAACCTCTCGGACAAGCAGGAGTCGTCC
TCGGCGCTGAAGCCCGGTGCCAGTAAGACCGGCGGCTGGCGGTTGTCGGACCTCAGCGCC
TCCGGCACGTTCGTCCGGAAGCTTATGTCGACGCATGGCCTGCAGTGGTACGGCGACATC
ATCAACAAGAACGCGTTCAAGGCGCTCTCGAAGGACTGGGAGTGCAACGTGATCCGACTA
GCCATGTACGTCGGGGAGGGGGGGTATGCGAGCAACCCAAGTATTAAGGAGAAGGTTATC
GAAGGGATCAAGCTCGCGATCGAGAACGACATGTACGTCATCGTCGACTGGCACGTGCTC
AATCCGGGGGACCCGAACGCAGAAATCTACAAGGGCGCGAAGGACTTCTTCAAGGAGATC
GCGACCTCGTTCCCGAACGACTACCACATCATCTACGAGCTGTGCAACGAACCGAACCCC
AACGAGCCCGGCGTCGAGAACTCCCTGGACGGTTGGAAGAAGGTGAAGGCCTATGCACAA
CCGATCATAAAGATGCTGCGCTCTCTCGGGAACCAGAACATCATCATCGTCGGTTCGCCA
AACTGGTCGCAGCGCCCCGACTTCGCGATCCAGGACCCGATCAACGACAAGAACGTGATG
TACTCGGTGCACTTCTACAGCGGCACCCACAAGGTGGACGGCTACGTGTTCGAGAACATG
AAGAACGCCTTCGAAAACGGCGTGCCGATCTTCGTGTCGGAGTGGGGCACGTCCCTGGCG
TCGGGCGACGGGGGGCCGTACCTCGACGAGGCGGATAAGTGGCTGGAGTACCTGAACTCG
AACTACATCTCGTGGGTGAACTGGAGCCTCTCCAATAAGAACGAGACCTCCGCGGCATTC
GTGCCCTACATCAACGGCATGCACGACGCCACCCCGCTGGACCCCGGAGACGATAAGGTG
TGGGACATCGAGGAACTGTCCATCTCCGGTGAGTATGTGAGGGCCCGTATCAAAGGGATC
GCCTACCAGCCGATCAAACGCGACAACAAGATCAAGGAGGGCGAGAATGCGCCCCTTGGC
GAGAAGGTCCTGCCGTCCACGTTCGAAGACGACACCCGGCAGGGCTGGGATTGGGACGGT
CCCTCCGGTGTTAAAGGACCGATCACCATCGAATCGGCCAACGGATCCAAGGCGCTCTCA
TTCAACGTCGAATACCCCGAGAAGAAGCCGCAGGACGGGTGGGCAACGGCGGCCCGGCTC
ATTCTCAAAGACATCAACGTCGAACGCGGCAACAACAAGTACCTCGCGTTCGACTTCTAC
CTGAAACCTGACCGGGCCAGCAAGGGCATGATTCAGATCTTCTTGGCGTTCTCGCCGCCG
TCGTTGGGTTACTGGGCCCAGGTGCAGGATTCGTTCAACATCGACCTGGCAAAGCTGAGC
TCAGCGAAGAAGATCGAGGACCGCATATACAAGTTCAATGTCTTCTTCGATCTGGACAAG
ATCCAGGACAACAAGGTCCTCAGCCCGGACACCCTGCTCCGCGACATCATCGTCGTCATC
GCCGACGGCAACTCCGACTTCAAGGGGAAGATGTACATCGACAACGTGCGGTTCACCAAC
ATCCTGTTCGAGGACATCAACTTCGAAAACTCGCTGTACGACGTGATCGACCTCGAGCAT
CACCATCACCATCAC**TGA**

Protein Sequence (Sec signal peptide underlined)

<u>MKRSISIFITCLLITLLTMGGMIASPAS</u>AAGTKTPVAKNGQLSIKGTQLVNRDGKAVQLK
GISSHGLQWYGEYVNKDSLKWLRDDWGITVFRAAMYTADGGYIDNPSVKNKVKEAVEAAK
ELGIYVIIDWHILNDGNPNQNKEKAKEFFKEMSSLYGNTPNVIYEIANEPNGDVNWKRDI
KPYAEEVISVIRKNDPDNIIIVGTGTWSQDVNDAADDQLKDANVMYALHFYAGTHGQFLR
DKANYALSKGAPIFVTEWGTSDASGNGGVFLDQSREWLKYLDSKTISWVNWNLSDKQESS
SALKPGASKTGGWRLSDLSASGTFVRKLMSTHGLQWYGDIINKNAFKALSKDWECNVIRL

AMYVGEGGYASNPSIKEKVIEGIKLAIENDMYVIVDWHVLNPGDPNAEIYKGAKDFFKEI
ATSFPNDYHIIYELCNEPNPNEPGVENSLDGWKKVKAYAQPIIKMLRSLGNQNIIIVGSP
NWSQRPDFAIQDPINDKNVMYSVHFYSGTHKVDGYVFENMKNAFENGVPIFVSEWGTSLA
SGDGGPYLDEADKWLEYLNSNYISWVNWSLSNKNETSAAFVPYINGMHDATPLDPGDDKV
WDIEELSISGEYVRARIKGIAYQPIKRDNKIKEGENAPLGEKVLPSTFEDDTRQGWDWDG
PSGVKGPITIESANGSKALSFNVEYPEKKPQDGWATAARLILKDINVERGNNKYLAFDFY
LKPDRASKGMIQIFLAFSPPSLGYWAQVQDSFNIDLAKLSSAKKIEDRIYKFNVFFDLDK
IQDNKVLSPDTLLRDIIVVIADGNSDFKGKMYIDNVRFTNILFEDINFENSLYDVIDLEH
HHHHH-
Theoretical pI/Mw: **5.36 / 94880.91**

Cel5AC from *Bacillus subtilis* Optimized Sequence Length: 1014, GC%: 59%

ATGAAGCGCTCCATCTCGATCTTCATCACCTGCCTGCTGATCACGCTCCTCACGATGGGC
GGCATGATCGCGTCCCCCGCGAGCGCCGCCGGCACCAAGACTCCGGTGGCGAAAAACGGG
CAGTTGAGCATCAAGGGCACACAGCTAGTGAACCGCGACGGCAAGGCGGTGCAACTGAAG
GGTATCTCGTCCCACGGGCTCCAGTGGTACGGAGAGTACGTAAATAAGGATAGCCTGAAG
TGGCTCCGTGATGATTGGGGTATCACCGTCTTCCGCGCAGCGATGTACACCGCCGACGGA
GGATACATTGATAATCCCTCGGTCAAGAACAAGGTCAAGGAGGCCGTCGAAGCCGCGAAG
GAATTGGGCATCTACGTCATCATCGACTGGCACATCCTCAACGACGGGAACCCGAACCAG
AACAAAGAGAAGGCCAAGGAGTTCTTCAAGGAGATGAGCTCCCTCTACGGCAACACCCCC
AACGTTATCTACGAGATCGCCAACGAGCCCAATGGTGACGTGAATTGGAAGCGAGACATC
AAGCCGTACGCCGAGGAGGTCATCTCCGTCATCCGCAAGAATGACCCGGACAACATCATC
ATCGTGGGCACTGGCACGTGGTCCCAGGACGTCAACGACGCCGCGGACGACCAGCTCAAA
GACGCGAACGTCATGTACGCCCTGCACTTCTACGCCGGAACGCACGGCCAGTTTCTTCGG
GACAAGGCCAACTACGCCCTGTCGAAGGGCGCCCCCATCTTCGTCACCGAGTGGGGAACC
AGTGATGCGAGCGGAAATGGCGGTGTCTTCCTCGACCAGTCGCGGGAATGGCTGAAGTAT
CTCGACAGCAAGACGATCTCGTGGGTCAACTGGAACCTCTCGGACAAGCAGGAGTCGTCC
TCGGCGCTGAAGCCCGGTGCCAGTAAGACCGGCGGCTGGCGGTTGTCGGACCTCAGCGCC
TCCGGCACGTTCGTCCGGAAGCTTAGATCTCTCGAGCATCACCATCACCATCACTGA

Protein Sequence (Sec signal peptide underlined)

MKRSISIFITCLLITLLTMGGMIASPASAAGTKTPVAKNGQLSIKGTQLVNRDGKAVQLK
GISSHGLQWYGEYVNKDSLKWLRDDWGITVFRAAMYTADGGYIDNPSVKNKVKEAVEAAK
ELGIYVIIDWHILNDGNPNQNKEKAKEFFKEMSSLYGNTPNVIYEIANEPNGDVNWKRDI
KPYAEEVISVIRKNDPDNIIIVGTGTWSQDVNDAADDQLKDANVMYALHFYAGTHGQFLR
DKANYALSKGAPIFVTEWGTSDASGNGGVFLDQSREWLKYLDSKTISWVNWNLSDKQESS
SALKPGASKTGGWRLSDLSASGTFVRKLRSLEHHHHHH-

Theoretical pI/Mw: 7.79 / 37522.30

Cel48 from *Thermobifida fusca* Original Sequence Length: 2958 , GC%: 67%

ATGAGATCGTTACTGTCTCCCCGGCGCTGGCGCACGCTGGCCTCGGGGGCGCTCGCAGCG
GCCCTGGCCGCCGCTGTACTCTCCCCCGGCGTCGCGCACGCCGCCGTCGCCTGCTCGGTG
GACTACGACGACTCCAACGACTGGGGTAGCGGGTTCGTCGCCGAAGTCAAGGTGACCAAC
GAAGGCAGCGACCCCATCCAGAACTGGCAAGTAGGCTGGACCTTCCCCGGTAACCAGCAG
ATCACCAACGGCTGGAACGGCGTGTTCAGCCAGAGCGGCGCCAACGTCACCGTCCGCTAC
CCGGACTGGAACCCCAATATCGCCCCCGGAGCCACCATCTCCTTCGGCTTCCAGGGCACC
TACAGCGGCTCCAACGACGCCCCGACCAGCTTCACCGTCAACGGCGTCACCTGCAGCGGA
TCCCAGCCCGCCAACCTGCCGCCCGATGTCACCCTGACATCCCCGGCCAACAACTCGACC
TTCCTGGTCAACGACCCGATCGAGCTGACCGCGGTCGCCTCCGACCCCGACGGCTCGATC
GACCGGGTGGAATTCGCCGCCGACAACACCGTCATCGGCATCGACACCACCTCCCCCTAC
AGCTTCACCTGGACGGACGCTGCCGCCGGCTCCTACTCGGTGACCGCGATCGCCTACGAC
GACCAGGGAGCCAGGACCGTCTCCGCTCCCATCGCCATCCGAGTGCTGGACCGGGCCGCC
GTCATCGCCTCACCGCCCACCGTCCGCGTGCCGCAGGGCGGCACCGCCGACTTCGAGGTG
CGGCTGTCCAACCAGCCCTCCGGCAACGTCACGGTCACCGTGGCGCGCACGTCGGGCAGC
TCCGACCTGACCGTCTCCAGCGGCTCCCAACTCCAGTTCACCTCCAGCAACTGGAACCAG
CCGCAGAAGGTGACCATCGCCTCCGCTGACAACGGCGGAAACCTGGCCGAGGCGGTCTTC
ACCGTCAGCGCCCCCGGCCACGACTCGGCCGAGGTGACGGTCCGGGAGATCGACCCGAAC
ACCAGCTCCTACGACCAGGCCTTCCTGGAGCAGTACGAGAAGATCAAGGACCCCGCCAGC
GGCTACTTCCGCGAATTCAACGGGCTCCTGGTCCCCTACCACTCGGTGGAGACCATGATC
GTCGAGGCTCCGGACCACGGCCACCAGACCACGTCCGAGGCGTTCAGCTACTACCTGTGG
CTGGAGGCGTACTACGGCCGGGTCACCGGTGACTGGAAGCCGCTCCACGACGCCTGGGAG
TCGATGGAGACCTTCATCATCCCCGGCACCAAGGACCAGCCGACCAACTCCGCCTACAAC
CCGAACTCCCCGGCGACCTACATCCCCGAGCAGCCCAACGCTGACGGCTACCCGTCGCCT
CTCATGAACAACGTCCCGGTGGGTCAAGACCCGCTCGCCCAGGAGCTGAGCTCCACCTAC
GGGACCAACGAGATCTACGGCATGCACTGGCTGCTCGACGTGGACAACGTCTACGGCTTC
GGGTTCTGCGGCGACGGCACCGACGACGCCCCCGCCTACATCAACACCTACCAGCGTGGT
GCGCGCGAGTCGGTGTGGGAGACCATTCCGCACCCGTCCTGCGACGACTTCACGCACGGC
GGCCCCAACGGCTACCTGGACCTGTTCACCGACGACCAGAACTACGCCAAGCAGTGGCGC
TACACCAACGCCCCCGACGCTGACGCGCGGGCCGTCCAGGTGATGTTCTGGGCGCACGAA
TGGGCCAAGGAGCAGGGCAAGGAGAACGAGATCGCGGGCCTGATGGACAAGGCGTCCAAG
ATGGGCGACTACCTCCGGTACGCGATGTTCGACAAGTACTTCAAGAAGATCGGCAACTGC
GTCGGCGCCACCTCCTGCCCGGGTGGCCAAGGCAAGGACAGCGCGCACTACCTGCTGTCC
TGGTACTACTCCTGGGGCGGCTCGCTCGACACCTCCTCTGCGTGGGCGTGGCGTATCGGC
TCCAGCTCCTCGCACCAGGGCTACCAGAACGTGCTCGCTGCCTACGCGCTCTCGCAGGTG
CCCGAACTGCAGCCTGACTCCCCGACCGGTGTCCAGGACTGGGCCACCAGCTTCGACCGC
CAGTTGGAGTTCCTCCAGTGGCTGCAGTCCGCTGAAGGTGGTATCGCCGGTGGCGCCACC
AACAGCTGGAAGGGAAGCTACGACACCCCGCCGACCGGCCTGTCGCAGTTCTACGGCATG
TACTACGACTGGCAGCCGGTCTGGAACGACCCGCCGTCCAACAACTGGTTCGGCTTCCAG
GTCTGGAACATGGAGCGCGTCGCCCAGCTCTACTACGTGACCGGCGACGCCCGGGCCGAG
GCCATCCTCGACAAGTGGGTGCCGTGGGCCATCCAGCACACCGACGTGGACGCCGACAAC
GGCGGCCAGAACTTCCAGGTCCCCTCCGACCTGGAGTGGTCGGGCCAGCCTGACACCTGG
ACCGGCACCTACACCGGCAACCCGAACCTGCACGTCCAGGTCGTCTCCTACAGCCAGGAC
GTCGGTGTGACCGCCGCTCTGGCCAAGACCCTGATGTACTACGCGAAGCGTTCGGGCGAC
ACCACCGCCCTCGCCACCGCGGAGGGTCTGCTGGACGCCCTGCTGGCCCACCGGGACAGC
ATCGGTATCGCCACCCCCGAGCAGCCGAGCTGGGACCGTCTGGACGACCCGTGGGACGGC
TCCGAGGGCCTGTACGTGCCGCCGGGCTGGTCGGGCACCATGCCCAACGGTGACCGCATC
GAGCCGGGCGCGACCTTCCTGTCCATCCGCTCGTTCTACAAGAACGACCCGCTGTGGCCG
CAGGTCGAGGCACACCTGAACGACCCGCAGAACGTCCCGGCGCCGATCGTGGAGCGCCAC
CGCTTCTGGGCTCAGGTGGAAATCGCGACCGCGTTCGCAGCCCACGACGAACTGCTCGAG
CATCACCATCACCATCACTGA

Protein Sequence (Sec signal peptide underlined)

<u>MRSLLSPRRWRTLASGALAAALAAAVLSPGVAH</u>AAVACSVDYDDSNDWGSGFVAEVKVTN
EGSDPIQNWQVGWTFPGNQQITNGWNGVFSQSGANVTVRYPDWNPNIAPGATISFGFQGT
YSGSNDAPTSFTVNGVTCSGSQPANLPPDVTLTSPANNSTFLVNDPIELTAVASDPDGSI
DRVEFAADNTVIGIDTTSPYSFTWTDAAAGSYSVTAIAYDDQGARTVSAPIAIRVLDRAA
VIASPPTVRVPQGGTADFEVRLSNQPSGNVTVTVARTSGSSDLTVSSGSQLQFTSSNWNQ
PQKVTIASADNGGNLAEAVFTVSAPGHDSAEVTVREIDPNTSSYDQAFLEQYEKIKDPAS
GYFREFNGLLVPYHSVETMIVEAPDHGHQTTSEAFSYYLWLEAYYGRVTGDWKPLHDAWE
SMETFIIPGTKDQPTNSAYNPNSPATYIPEQPNADGYPSPLMNNVPVGQDPLAQELSSTY
GTNEIYGMHWLLDVDNVYGFGFCGDGTDDAPAYINTYQRGARESVWETIPHPSCDDFTHG
GPNGYLDLFTDDQNYAKQWRYTNAPDADARAVQVMFWAHEWAKEQGKENEIAGLMDKASK
MGDYLRYAMFDKYFKKIGNCVGATSCPGGQGKDSAHYLLSWYYSWGGSLDTSSAWAWRIG
SSSSHQGYQNVLAAYALSQVPELQPDSPTGVQDWATSFDRQLEFLQWLQSAEGGIAGGAT
NSWKGSYDTPPTGLSQFYGMYYDWQPVWNDPPSNNWFGFQVWNMERVAQLYYVTGDARAE
AILDKWVPWAIQHTDVDADNGGQNFQVPSDLEWSGQPDTWTGTYTGNPNLHVQVVSYSQD
VGVTAALAKTLMYYAKRSGDTTALATAEGLLDALLAHRDSIGIATPEQPSWDRLDDPWDG
SEGLYVPPGWSGTMPNGDRIEPGATFLSIRSFYKNDPLWPQVEAHLNDPQNVPAPIVERH
RFWAQVEIATAFAAHDELLEHHHHHH-

Theoretical pI/Mw: 4.48 / 107691.99

Table S4 BLAST results for *R. jostii* RHA1 beta-glucosidase genes

BLAST search on RHA1_ro01034 (accession Q0SHX5), showing >60% identity

Entry	Protein names	Identity
Q0SHX5	Beta-glucosidase (Rhodococcus jostii (strain RHA1)	100.0%
W8HGX8	Beta-glucosidase (Rhodococcus opacus PD630)	94.4%
A0A2S2BUS4	Glycoside hydrolase family 1 (Rhodococcus sp. S2-17)	79.3%
A0A1P8YJT3	Glycosyl hydrolase 1 family protein (Rhodococcus sp. MTM3W5.2)	72.9%
F1TJ69	Glycosyl hydrolase, family 1 (Rhodococcus hoagii ATCC 33707)	71.9%
A0A0D8HP97	Beta-glucosidase B (Rhodococcus sp. (strain AD45))	69.5%
A0A1X0ULY5	Glycoside hydrolase family 1 (Rhodococcus sp. 1168)	68.3%
A0A1G6Z0G3	Beta-glucosidase (Rhodococcus tukisamuensis)	68.1%
A0A541AZJ4	Glycoside hydrolase family 1 protein (Rhodococcus spelaei)	68.3%
A0A0K2YHL5	Beta-glucosidase (Rhodococcus sp. RD6.2)	68.4%
A0A4R7V7F6	Glycosyl hydrolase family 1 (Actinophytocola oryzae)	66.3%
A0A7W7QD21	Beta-glucosidase (Actinophytocola algeriensis)	65.3%
A0A1Q8BVF8	Glycoside hydrolase family 1 (Actinophytocola xanthii)	64.7%
A0A6B2E0J2	Glycoside hydrolase family 1 protein (Amycolatopsis sp. SID8362)	64.7%
A0A2S0KCK5	Glycoside hydrolase family 1 (Gordonia iterans)	61.0%
A0A4Q7JDR0	Glycoside hydrolase family 1 protein (Amycolatopsis suaedae)	64.2%
A0A059MPX9	Glycoside hydrolase family 1 (Rhodococcus aetherivorans)	65.9%
A0A6L7GMC1	Family 1 glycosylhydrolase (Gordonia sp. HNM0687)	63.0%
A0A4S8QJV5	Glycoside hydrolase family 1 protein (Glycomyces buryatensis)	61.4%
A0A3G8JG81	Beta-glucosidase (Gordonia insulae)	61.3%
V8CSA8	Glycoside hydrolase family 1 (Williamsia sp. D3)	58.7%
A0A2X4U2D0	Beta-glucosidase (Rhodococcus coprophilus)	63.1%
A0A344L466	Glycoside hydrolase family 1 (Amycolatopsis albispora)	61.1%
W5WEG0	Beta-glucosidase (Kutzneria albida DSM 43870)	60.4%
M0QPT4	Putative beta-glucosidase (Gordonia soli NBRC 108243)	60.9%

BLAST search on RHA1_ro02947 (accession Q0SCI4), showing >60% identity

Entry	Protein names	Identity
Q0SCI4	Beta-glucosidase (Rhodococcus jostii (strain RHA1)	100.0%
A0A6L9XUT9	Beta-glucosidase (Diaminobutyricibacter tongyo…)	77.0%
A0A0Q8UPN5	Beta-glucosidase (Leifsonia sp. Root227)	73.2%
A0A0Q5LM51	Beta-glucosidase (Leifsonia sp. Leaf336)	72.6%
A0A0M4M5G4	Beta-glucosidase (Arthrobacter sp. ERGS1:01)	69.9%
A0A4Q2L4P2	Beta-glucosidase (Agromyces albus)	68.1%
A0A0Q4HCP3	Beta-glucosidase (Agromyces sp. Leaf222)	67.2%
A0A3S4ANZ6	Beta-glucosidase (Labedella phragmitis)	66.9%
A0A2P8GW00	Beta-glucosidase (Labedella gwakjiensis)	64.5%
A0A4R2DRM9	Beta-glucosidase (Rhodococcus sp. SMB37)	67.6%
A0A2S3ZX96	Glycosyl hydrolase (Arthrobacter glacialis)	65.5%
V9XLW4	Beta-glucosidase (Rhodococcus pyridinivorans S…)	66.9%
A0A1H4LGA8	Beta-glucosidase (Microbacterium humi)	66.7%
A0A0Q8CKB3	Beta-glucosidase (Leifsonia sp. Root60)	65.9%
A0A0A1CXF7	Beta-glucosidase (Arthrobacter sp. PAMC 25486)	65.1%
A0A120I0V9	Glycosyl hydrolase (Microterricola viridarii)	63.8%
A0A2S5X205	Glycosyl hydrolase (Pseudoclavibacter sp. RFBA6)	64.3%
A0A5C1YFK1	Beta-glucosidase (Agromyces intestinalis)	64.7%
A0A166H979	Thermostable beta-glucosidase B (Rathayibacter tanaceti)	63.7%
A0A3M8AN19	Beta-glucosidase (Agromyces tardus)	62.0%
A0A543HUJ3	Beta-glucosidase (Humibacillus xanthopallidus)	64.0%
Z9JPU4	Beta-glucosidase (Brachybacterium phenoliresis…)	64.5%
A0A4Q2M7H4	Beta-glucosidase (Agromyces atrinae)	63.9%
A0A1E8CUT7	Glycosyl hydrolase (Humibacillus sp. DSM 29435)	63.3%
A0A543EUN1	Beta-glucosidase (Microbacterium kyungheense)	63.3%
A0A6G7Z2C5	Beta-glucosidase (Sanguibacter sp. HDW7)	62.8%
A0A3A5HB88	Beta-glucosidase (Nocardioides sp. K1W22B-1)	62.5%
A0A7Y9GLX7	Beta-glucosidase (Microbacterium immunditiarum)	63.8%
A0A0M2HFK9	Thermostable beta-glucosidase B (Microbacterium ketosireducens)	61.7%
A0A2N9JH15	Thermostable beta-glucosidase B (Micropruina glycogenica)	61.4%
A0A0M2HDD0	Thermostable beta-glucosidase B (Microbacterium trichotheceno…)	62.6%
A0A0B2A245	Beta-glucosidase (Microbacterium mangrovi)	62.5%
A0A0U4FKP6	Fn3_like domain-containing protein (Microbacterium sp. XT11**)**	61.6%
A0A4V2ZSL2	Beta-glucosidase (Arthrobacter terricola)	61.0%
A0A7X0KU95	Beta-glucosidase (Microbacterium thalassium)	62.4%
A0A1I4I0D6	Beta-glucosidase (Leifsonia sp. CL147)	61.6%
A0A0F0KEA0	Thermostable beta-glucosidase B (Microbacterium foliorum)	61.1%
A0A3L7APC9	Beta-glucosidase (Mycetocola lacteus)	60.4%
A0A0Q8LIE9	Beta-glucosidase (Microbacterium sp. Root180)	62.7%
A0A543BCG7	Beta-glucosidase (Microbacterium saperdae)	60.1%
W0Z818	Thermostable beta-glucosidase B (Microbacterium sp. C448)	61.9%
A0A1G8BHG9	Beta-glucosidase (Rhodococcus triatomae)	60.7%
A0A0T1WEJ2	Beta-glucosidase (Mycobacterium sp. Root135)	62.0%
A0A1T4TBD7	Beta-glucosidase (Marinactinospora thermotoler…)	61.2%
A0A0F2C6W3	Thermostable beta-glucosidase B (Microbacterium sp. SA39)	60.5%
A0A0F0L422	Thermostable beta-glucosidase B (Microbacterium azadirachtae)	61.0%
T5L4C2	Fn3_like domain-containing protein (Microbacterium maritypicum)	59.8%
A0A255G238	Glycosyl hydrolase (Propionibacteriaceae bacteri…)	62.3%
A0A1I6MDY7	Beta-glucosidase (Microbacterium sp. cf046)	60.9%
A0A543FRN3	Beta-glucosidase (Pseudonocardia cypriaca)	61.3%
A0A378TN84	Glycoside hydrolase family 3 domain protein (Mycolicibacterium tokaiense)	63.3%
A0A4P6ECI4	Beta-glucosidase (Microbacterium protaetiae)	60.2%
A0A4P8KT18	Beta-glucosidase (Microbacterium sp. RG1)	62.3%
A0A2G5PG11	Glycosyl hydrolase (Mycolicibacterium brumae)	60.9%
A0A562IM45	Beta-glucosidase (Modestobacter roseus)	60.5%

Entry	Protein names	Identity
A0A7Y9I5H4	Beta-glucosidase (Microlunatus parietis)	59.0%
F5XM35	Beta-glucosidase (Microlunatus phosphovorus (s…)	61.4%
A0A498CAB1	Beta-glucosidase (Microbacterium telephonicum)	60.7%
A0A5S9PJQ3	Thermostable beta-glucosidase B (Mycolicibacterium vanbaalenii)	60.8%
A0A1I2B5M9	Beta-glucosidase (Actinoplanes philippinensis)	60.0%
A0A7W5ADD5	Beta-glucosidase (Actinoplanes campanulatus)	60.3%
I0H1P8	Putative glycosyl hydrolase (Actinoplanes missouriensis (…)	60.1%
A0A0M2LFS5	Beta-glucosidase (Leucobacter sp. Ag1)	60.8%
A0A2U1TE32	Glycosyl hydrolase (Mycetocola zhujimingii)	62.2%
A0A0F0L3R0	Thermostable beta-glucosidase B (Microbacterium foliorum)	60.3%
A0A0J6VSU2	Thermostable beta-glucosidase B (Mycolicibacterium chubuense)	61.0%
A0A1H2BQF9	Beta-glucosidase (Actinoplanes derwentensis)	60.1%
A0A5J5KYB7	Beta-glucosidase (Kocuria coralli)	61.3%

Table S5. List of chromosomal *R. jostii* RHA1 genes referred to in this study

Gene ID (Uniprot ID)	Annotation ([a]Uniprot; [b]this work}	Size of encoded protein
**Beta-glucosidase genes**		
RHA1_ro01034 (Q0SHX5)	β-glucosidase BglA[a]	425 aa
RHA1_ro02947 (Q0SCI4)	β-glucosidase[a] BglB[b]	759 aa
**Quinic acid utilisation**		
RHA1_ro01367 (Q0SGZ5)	3-dehydroshikimate dehydratase[a]	644 aa
RHA1_ro01368 (Q0SGZ4)	3-dehydroquinate dehydratase[a]	146 aa
RHA1_ro01342 (Q0SH20)	Shikimate dehydrogenase (aroE1)[a] (probable quinate dehydrogenase[b])	304 aa
RHA1_ro01335<br>RHA1_ro01336	Protocatechuate 3,4-dioxygenase (pcaHG)[a]	
**Shikimate pathway genes**		
RHA1_ro03051 (Q0SC82)	3-dehydroquinate dehydratase[a]	155 aa
RHA1_ro01564 (Q0SGF9)	Shikimate dehydrogenase (aroE2)[a]	271 aa
RHA1_ro01853 (Q0SFM2)	Shikimate dehydrogenase (aroE3)[a]	300 aa
RHA1_ro07138 (Q0S0N4)	Shikimate dehydrogenase (aroE4)[a]	275 aa
RHA1_ro07140 (Q0S0N2)	Chorismate synthase (aroC)[a]	395 aa
RHA1_ro07141 (Q0S0N1)	Shikimate kinase (aroK)[a]	168 aa
RHA1_ro07142 (Q0S0N0)	3-Dehydroquinate synthase (aroB)[a]	393 aa

Figure S1. Vector Map for pTipQC2

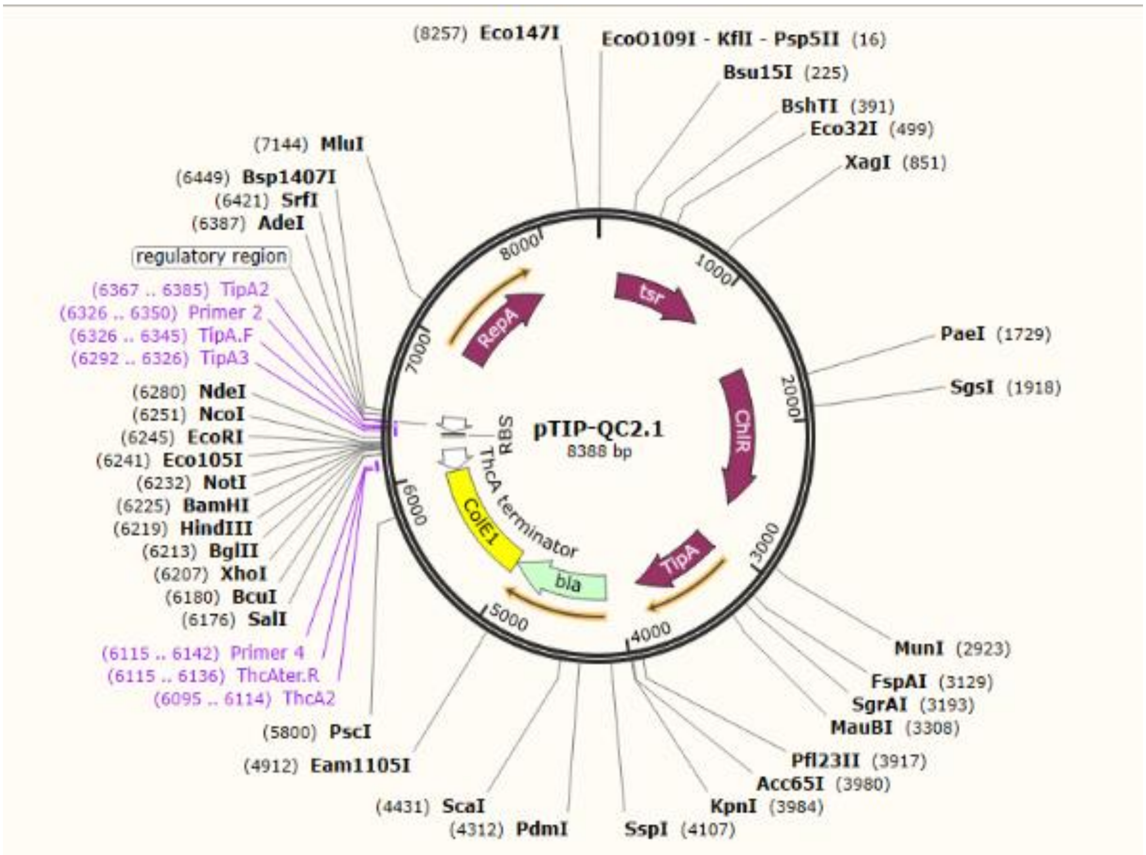

Figure S2. Agarose Gels for PCR Amplication, Digests and Colony PCR.

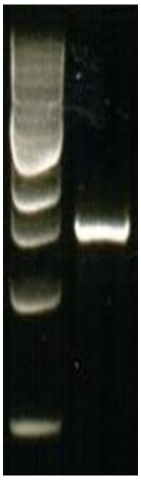

PCR of CenA

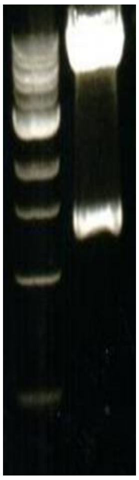

pTipCenA (NdeI-HindIII) [illegible]

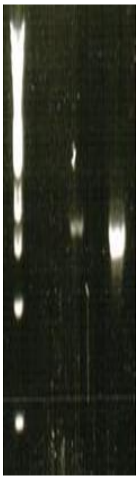

Colony PCR of ptipCenA from RHA1

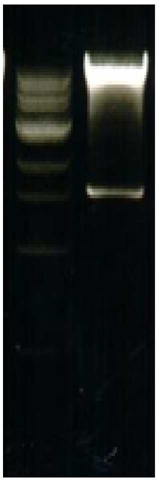

pTip Cel5A (Nde1-HindIII)

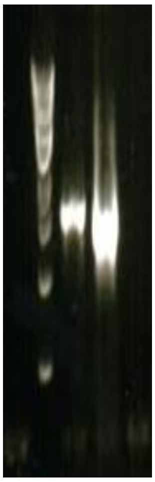

pTipCel5A Colony [illegible]

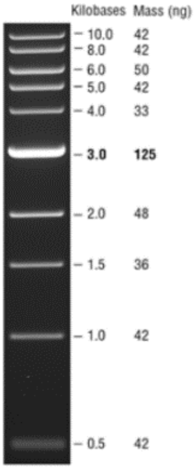

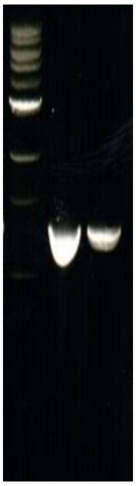

Cel6A

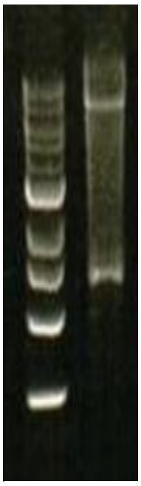

pTipCel6A (NdeI- [illegible]

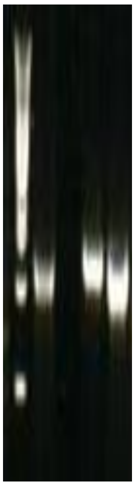

pTipCel6A

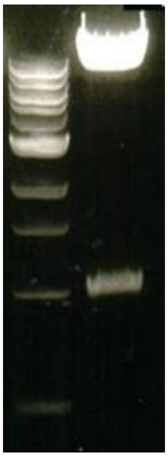

pTipCel5AC (Nde1-[illegible]

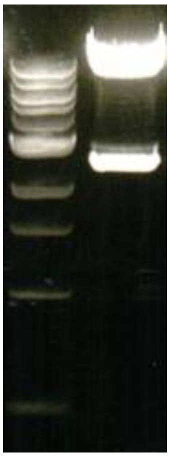

pTipCel5AC.D (Nde1-Xho1)

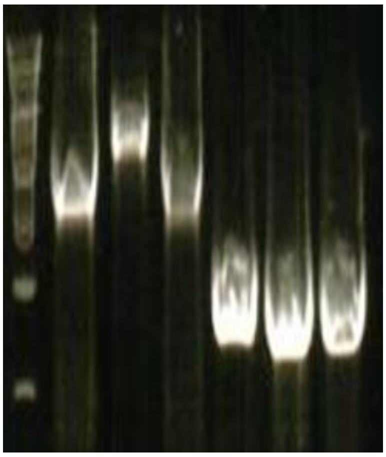

Lane 1,2,3 pTip Cel5AC and Lane 4,5,6 pTip Cel5ACD

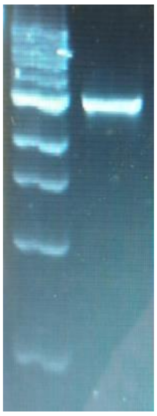

PCR of Cel48A

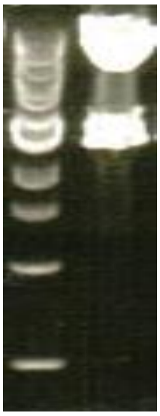

pTipCel48A (Nde1- [illegible]

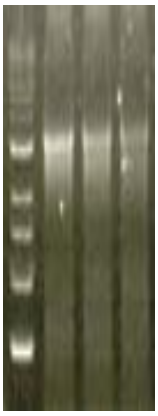

Colony PCR of [illegible]

Figure S3. Congo Red Staining of *R. jostii* pTipQC2-cellulase constructs.

A. Recombinant strains grown on M9 agar plates containing 0.5% carboxymethylcellulose and 0.1% glucose for 3 days at 30 °C, supplemented with thiostrepton after 24 hr (see Materials & Methods).

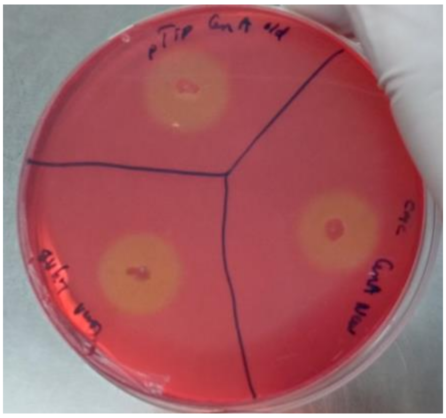

CMCase assay of pTip CenA and LigAB

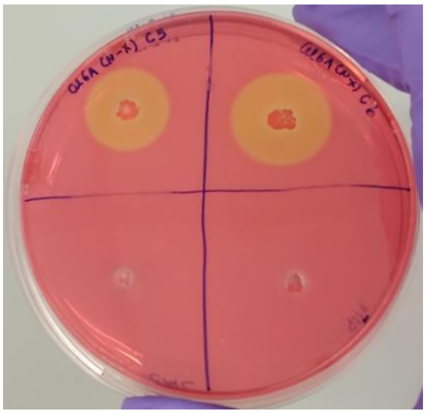

CMCase assay of pTip Cel6A

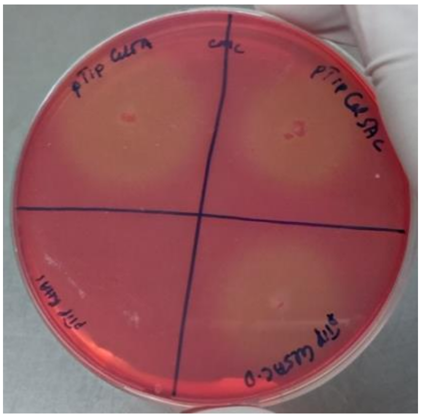

Congo Red Staining of Cel5A, Cel5AC and Cel5ACD

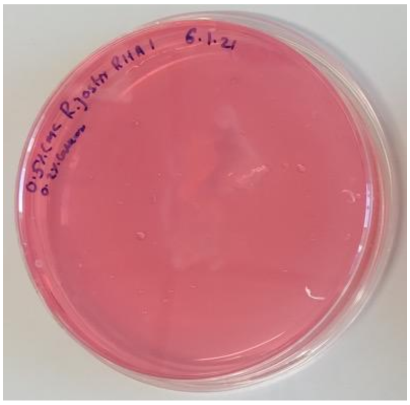

RHA1 wt

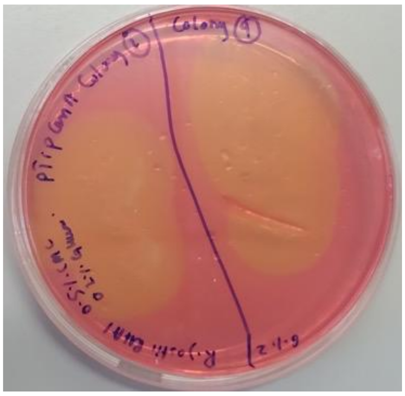

PTipcenA in RHA1 wt

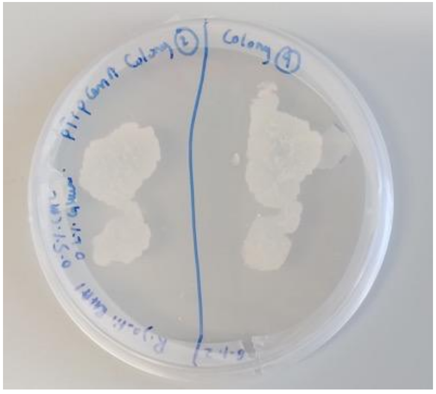

PTipcenA Before staining

B. Growth on M9 agar plates containing 0.5% carboxymethylcellulose (without glucose) for 3 days at 30 ^{o}C, supplemented with thiostrepton after 24 hr.

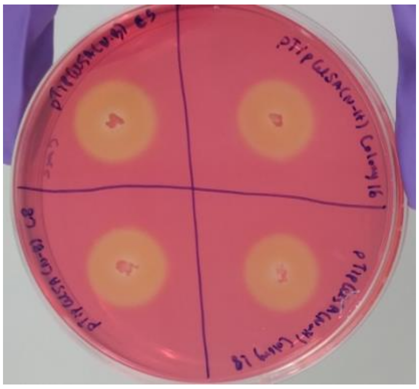

R. jostii pTipQC2-cel5A

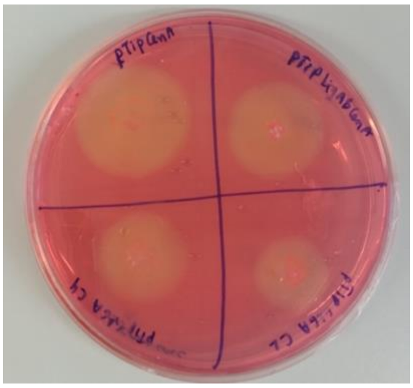

R. jostii pTipQC2-cenA and *R. jostii* pTipQC2-cel6A

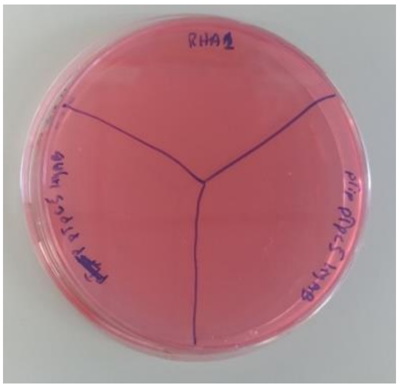

Wild-type *R. jostii* RHA1 and *R. jostii* pcaHG::ligAB
